# Mapping the climate and agronomic digital advisory services landscape in West and Central Africa: A step towards making food systems productive and climate resilient

**DOI:** 10.1371/journal.pone.0338010

**Published:** 2025-12-03

**Authors:** Manjari Singh, Mathieu Ouedraogo, Daniel Jimenez, Tiffany Talsma, Adama Ouedraogo, Desire Kagabo, Julian Ramirez, Peter Laderach

**Affiliations:** 1 Climate action, International Center for Tropical Agriculture (CIAT), Dakar, Senegal; 2 Digital Inclusion, Bioversity International, Versailles, France; 3 Digital Inclusion, Bioversity International, Accra, Ghana; 4 Institut de l’Environnement et de Recherches Agricoles (INERA), Bobo-Dioulasso, Burkina Faso; 5 Climate action, International Center for Tropical Agriculture (CIAT), Nairobi, Kenya; 6 Climate action, International Center for Tropical Agriculture (CIAT), Pretoria, South Africa; 7 Climate action, International Center for Tropical Agriculture (CIAT), Rome, Italy; Makerere University College of Natural Sciences, UGANDA

## Abstract

This study explores and examines the availability of digital agricultural solutions (DAGs) to enhance agricultural resilience in the face of climate change. The countries under study—Burundi, Ivory coast, Democratic Republic of Congo (DRC), Ghana, Nigeria, and Rwanda—display varying levels of vulnerability, suffering from low adaptive capacity to address climate impacts. The study examines available DAGs to support farmers, including mobile-based platforms providing weather and agronomic advice, market information, and financial services. We highlight the diverse needs and challenges faced by three key user groups of digital agro-climatic services: farmers, service providers, and policymakers. All stakeholders require tailored approaches to improve adoption and impact. We employed a mixed-method approach that combined literature review with semi-structured interviews to identify existing digital solutions in agriculture. Next, we produced country-specific reports to assess the current state of agronomic digital advisory services, their users, and challenges. Ghana, Nigeria, and Rwanda have made notable strides in delivering these services, though challenges such as inadequate infrastructure, high internet costs, and gender disparities hinder their wider adoption. Additionally, data fragmentation and lack of harmonization across platforms pose significant barriers to optimizing these digital solutions. We discuss the role of public extension services and policy frameworks in fostering digital transformation in agriculture, with an emphasis on the need for better data harmonization to improve decision-making. The study underscores the importance of integrating digital technologies with stronger policies, improved infrastructure, and greater inclusivity to support climate-resilient agricultural practices in the region.

## 1. Introduction

The net importer status of many African countries, amidst ongoing global conflicts, food crisis, and climate change, is a growing concern, especially for their rapidly increasing population. Food production in these countries remains consistently below average, with several key challenges impeding their progress. Limited access to resources—inputs (quality seeds, fertilizers, water), agricultural machinery, and credit during the planting season, combined with rainfed farming on a highly fragmented land holdings—contribute to challenges in farm mechanization and lowered crop production [1–2]. Additional challenges include inadequate disease control and surveillance systems [3], climatic constraints, poor market access due to substandard road infrastructure and insecurity, limited storage and processing facilities, high levels of rural outmigration, and price fluctuations [4]. These challenges are widespread, especially among smallholder farmers.

Nevertheless, food system productivity of these smallholder farmers can be increased sustainably by building resilience to the shocks of climate variability. Agricultural production resilience requires growing crops under optimal management conditions and adopting scientifically recommended agronomic practices to minimize the impact of biotic (pests, diseases) and abiotic (extreme weather) stress. Crops grown under optimal conditions yield well and ensure food security. To manage farms well, farmers need information on suitable crops, seed rates, irrigation timing, fertilizer schedules, pest control, and market data. Agro-advisories provide these information, along with weather forecast interpretation, supporting effective decision-making.

Given the importance of the agricultural sector in the national economy and the increasing challenges faced by farmers, several international organizations have been working to develop digital technologies that provide agronomic and climate advisory services to producers across various value chains. Partnership include the local government, telephone companies, and NGOs. During the past decade, a range of digital tools have been developed to assist and improve the extension services. Specialists design interventions tailored to the needs of different categories of farmers. For example, providers offer fertilizer recommendations [5], agronomic package of practices, climate advisory services [6], pest and disease surveillance [7], and other information services through various digital platforms. Mobile phone ownership and internet access play a key role in accessing these platforms and information services. The state of infrastructure development and other requirements for the successful implementation of digital agricultural services, however, varies across Africa.

This synthesis report, therefore, summarizes existing digital agricultural solutions (DAGs) of six countries in West and Central Africa. We assess socio-economic conditions, the state of agriculture, the information and communication technology infrastructure (if any), the tools available for improving access to financial and insurance services, and the user base. Agricultural information services are important in helping farmers to buffer investment, yield, and livelihood losses as a result of climatic shocks. Furthermore, we identified the challenges in implementing DAGs in the region, while also examining key aspects such as user research, the inclusivity of target users in decision-making, and data harmonization and management. The research aims to identify existing gaps in services farmers, to support research interventions and policy decision, and to study the impact of information services on the farming community and the food security in these countries.

## 2. Materials and methods: data sources, description, and empirical strategy

### 2.1. Geographic scope

**Fig 1:**
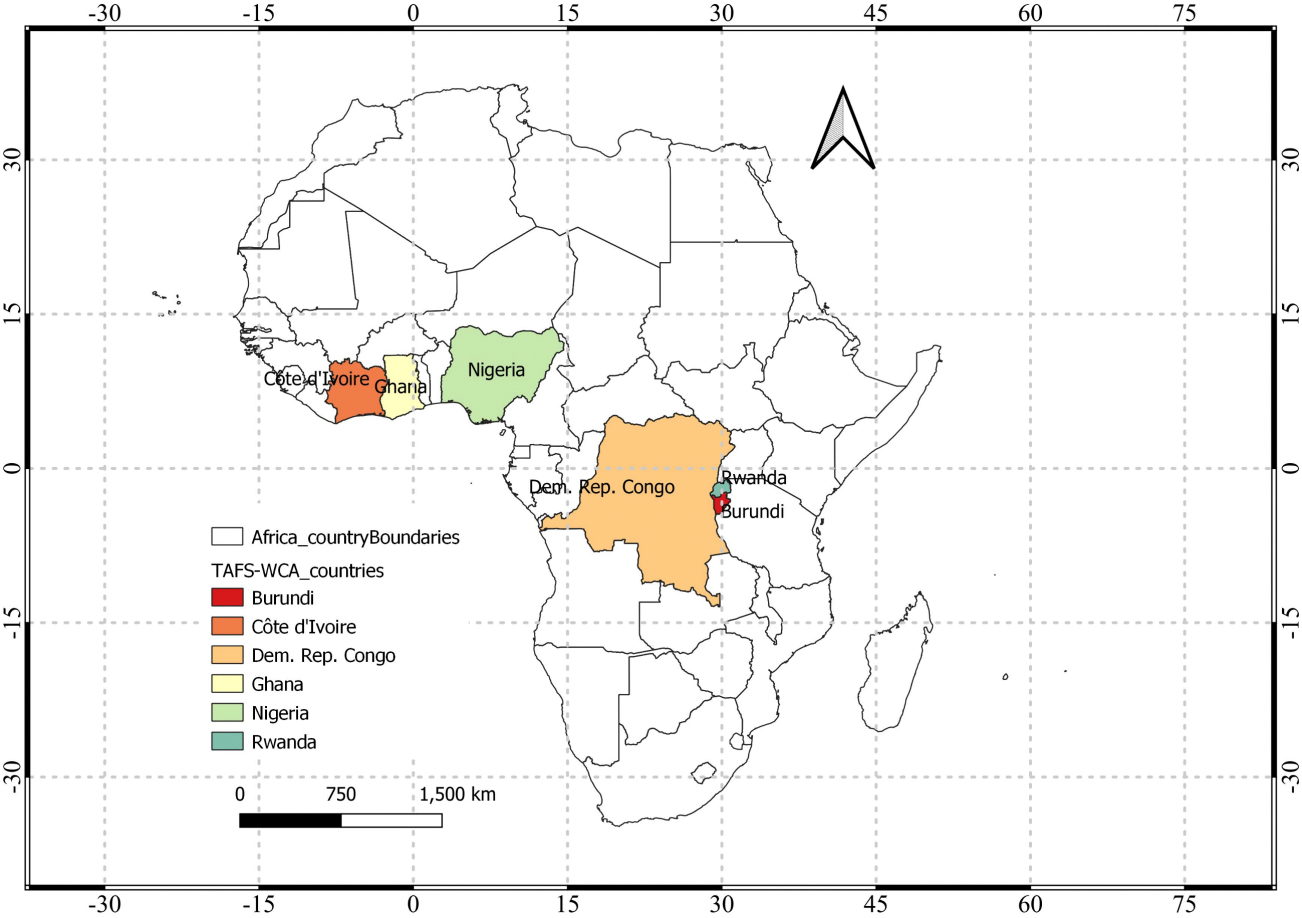
Administrative boundary of six countries selected for mapping under project “Transforming Agrifood Systems in West and Central Africa (TAFS-WCA)”.

Our work focuses on six countries of West and Central Africa (WCA) during 2021−22: Burundi, Ivory coast (Côte d’Ivoire in French; CI), the Democratic Republic of Congo (DRC), Ghana, Nigeria, and Rwanda (Fig 1).

### 2.2. Socio-economic conditions

The countries selected for this study are home to millions of people, with Nigeria being the most populous country [8]. Countries such as Rwanda and Burundi, although they are relatively smaller in area, are inhabited by over twenty-five million people (Table 1). In terms of income level, the countries were classified as *low-income* to *low-to-middle* income countries. The majority of the inhabitants were living in rural areas. More than 80% of the population of Burundi and Rwanda lives in rural areas, which explains their limited access to electricity, sanitation, and good quality drinking water. Large countries like the DRC—with only 15% of its land area suitable for agriculture [9], and where only 10% of this is currently being used [10]—face enormous challenges to reach out to their rural population to alleviate malnutrition and poverty. According to the Global Hunger Index [11], the state of hunger was *moderate* in Ghana, *serious* in Ivory coast, Nigeria and Rwanda, and *alarming* in Burundi and DRC (Table 1).

**Table 1:** Status of socio-economic variables in different countries.

Country	Rwanda	Ivory coast	Burundi	Nigeria	DRC	Ghana
**Socio-economic variables**						
Income level	low	Low – middle	low	Low-middle	Low	Low-middle
BPL population	52%	39.5%	72.90%	31% (72% farmers)	72%	23.40%
Population (million)	13.2	27	12.5	213.4	95.9	31.7
Access to drinking water	60%	76.6	83% in urban areas (60% rural)	68	52	~96 (74) % in urban (rural) areas
Contribution (%) of agriculture in GDP	27	20	27.57	24.4%	19.40	18.78
Contribution to employment (%)	56% (66.6% rural pop)	48%	NA	39% (55% rural pop)	60%	42%
Rural population	83.00	47.80	86.00	48.00	54.36	42.00
Arable land	51.4	11	49.5	40.5	6	20.7
Agricultural land	81.3	73.9	81.9	75.4	15	55.4
Forest area (% of total land area	11.2	8.6	10.9	23.6	55.2	35.1
**Food-security situation over the last decade**						
Status of country	Importer	Importer	NA	Importer	Importer	Importer
Malnutrition rate (% of children under 5 years)	35%	NA	52%	5 to 30%**	40%	19% (33% in northern region)
Rank of countries ***	96	86	NA	109	122	62
Level of hunger based on GHI	Serious	Serious	Alarming	Serious	Alarming	Moderate

* Compiled by the authors from different sources.

** 53% of the population in Nigeria are moderately food insecure and 26% are severely food insecure.

*** Rank among 125 countries, according to the Global Hunger Index (GHI).

Agriculture remains economically important for these countries, contributing between 18 and 28% to these country’s GDP (Table 1). In the past decade, however, with the increasing contribution of industrial and service sectors, agriculture’s contribution to the total GDP has fallen sharply. The contribution of agriculture to GDP was highest in Burundi, where cash crops such as coffee, cotton, tea, and palm oil account for about 90% of the country’s export. The area under cultivation has increased in most of these countries. However, extensive deforestation resulted in a sharp decline in the total forest area. Deforestation was relatively higher in Ivory coast and Ghana.

### 2.3. Climate

**Fig 2:**
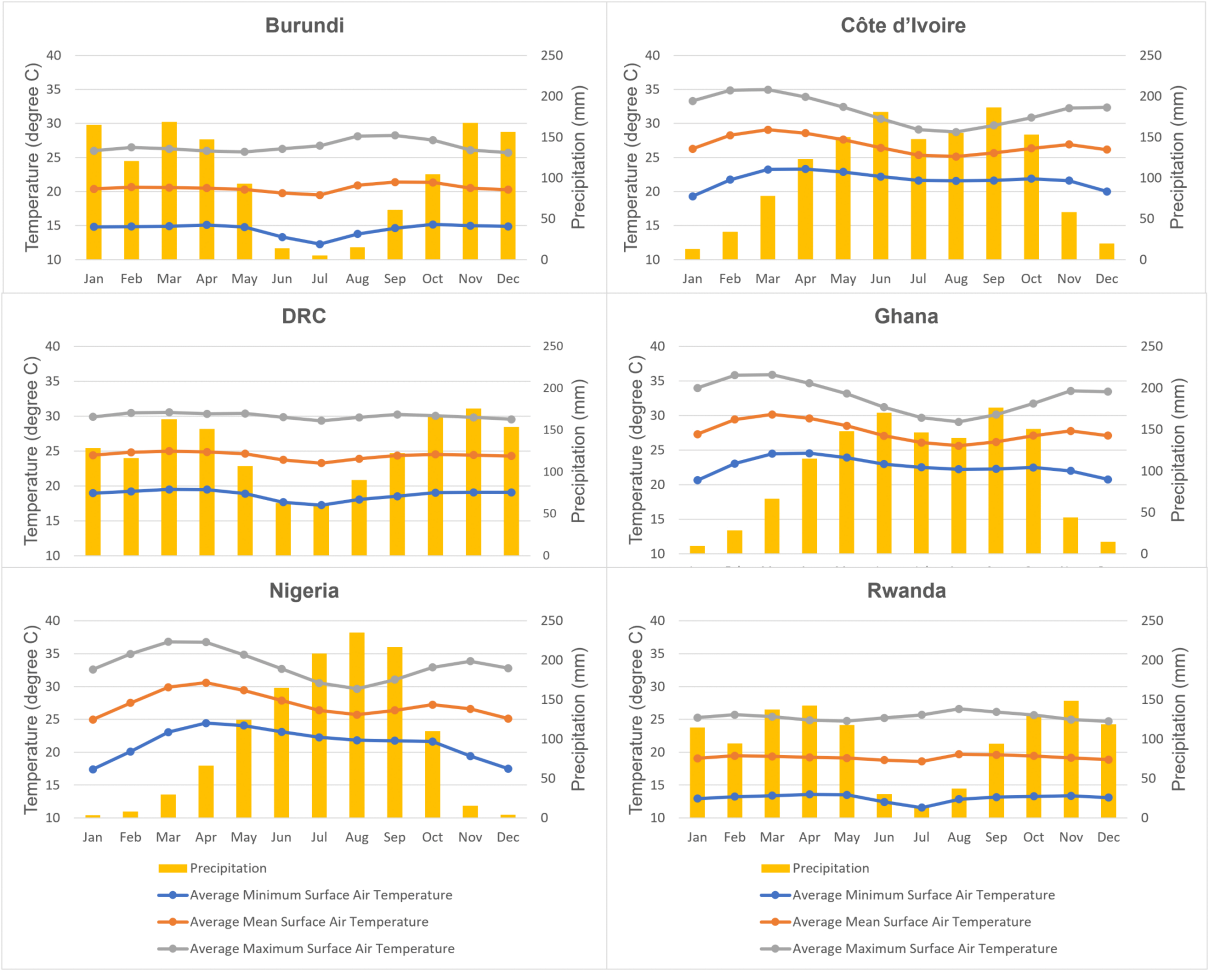
Monthly climatology (1991–2022) for precipitation, minimum, maximum, and average temperature in the six countries of wca (data source: world bank [12]).

Climate variables show great heterogeneity at both spatial and temporal scales. At the national level, the temporal distribution of rainy season is unimodal in Ghana, Nigeria, and Ivory coast (Fig 2). Rainfall seasonality is bimodal in Burundi, Rwanda, and the DRC. In West-African countries, the rainy season extends from April to November. In contrast, Burundi, Rwanda, and the DRC have four seasons: *long rainy season* (March−May); *short rainy season* (Sept−Nov); *long dry season* (June−Aug); and *short dry season* (Dec − Feb). Mean monthly surface air temperatures show little fluctuation in Rwanda, Burundi, and the DRC across the year. However, these curves showed greater variability in West African countries, exhibiting a conspicuous dip during the rainy season.

### 2.4. Status of farming and food security

Smallholder farmers in the region—relying on rainfed cultivation, grown with limited access to quality seeds, fertilizers, agrochemicals, or credit for machinery—suffer from low productivity [13]. Contributing factors include: (1) low adoption of improved seeds and technologies; (2) unmanaged pests, diseases, and weeds; (3) limited use of agrochemicals; (4) inadequate agricultural extension services; and (5) lack of information on weather, markets, and credit. While Ivory coast has high adoption of improved seeds for rice, maize, and cash crops, climate variability significantly impacts crop performance.

In the last decade, the agriculture sector has grown rapidly. Cash crops—including cocoa, tea, cashew nuts, palm oil, and natural rubber—drove most of this growth. The area farmed and production of staple crops such as maize, beans, corn, cassava, sweet potatoes, have also increased in the last decade, without any significant increase in yield. In contrast, the yield of bananas increased with a simultaneous decrease in area. Overall, the six study countries are net importers of foodgrains. Food imports accounts for around 18% of the total import, the majority of which are cereals (16.5–35%). Higher dependency on the global market for food grains and other key commodities makes these countries susceptible to sharp increases or fluctuations in world commodity prices.

### 2.5. Methodology

This study employed a mixed-method approach, that combined a literature review with semi-structured interviews to assess the landscape of climate and agronomic digital advisory services in six countries. The methodology consisted of the following steps:

**Step 1. Literature review and synthesis:** The first step involved conducting a comprehensive literature review to identify existing digital solutions in agriculture, focusing on climate and environmental risk management. This review aimed to collect and synthesize results from previous studies, reports, and relevant publications on digital innovations that support agricultural resilience in the face of climate change. Key themes reviewed include the types of technologies being used, the platforms and analytical techniques deployed, and the overall impact of digital services on decision-making in the agricultural sector. The literature review also explored cross-cutting issues such as data privacy, harmonization, and institutional capacity.

Our methodology and review relied on the Digital Agri Hub Dashboard (https://digitalagrihub.org/web/guest/dashboard). This online platform allowed us to identify and profile top-performing digital agriculture platforms in each target country. For each platform, the dashboard provides standardized, up-to-date information, including: (i) Number of users, (ii) Types of services offered (e.g., weather advisories, agronomic tips, market prices), (iii) Delivery channels (e.g., SMS, mobile apps, call centers, radio) and (iv) Geographic coverage (national, sub-national, or regional reach). This tool ensured a consistent and comparative assessment across countries.

To complement the use of Digital Agri Hub, a diverse range of online repositories and institutional websites were consulted, including: the Alliance Bioversity International & CIAT (https://alliancebioversityciat.org/publications-data): CTA (https://www.cta.int/fr/digitalisation), IITA (https://www.iita.org/), AGRA (https://agra.org/), AfricaRice (https://www.africarice.org/riceadvice), GSMA (https://www.gsma.com/r/digital-agriculture-maps/), WMO (https://public.wmo.int/en/our-mandate/weather), IFAD (https://www.ifad.org/), FEWSNET (https://fews.net/), Go Africa Online (https://www.goafricaonline.com/). We also used Country-specific portals such as: (i) https://agriculture.gouv.ci/ for Ivory coast; https://minagri.gouv.cd/ for DRC; etc. Social media platforms were also used to discover informal or emerging services.

A mixed search strategy was employed for the literature review. For quick mapping, search queries such as “Top 100 Digital Agriculture Services + [Country]” were used. The tilde symbol (~) was used to uncover related results. Online directories like Go Africa Online were also used to identify private actors. Only services with an online footprint were included. Offline-only services (e.g., USSD without documentation) were excluded. Regional or global initiatives active in the countries were included when relevant. The core keywords include: “climate digital services” and “agronomic digital advisory services”. These were combined with specific country names (e.g., “+Côte d’Ivoire”, “+Burundi”, “+DRC”, “+Ghana” “+Rwanda” “+Nigeria”) to target geographically relevant results. Given the prominent role of private sector actors such as startups, additional terms like “startup”, “digital platform”, “agritech” were also incorporated to identify non-governmental service providers.

The literature review provided a foundational understanding of the digital advisory landscape in each country but also revealed gaps and context-specific information that required further exploration. To address these gaps and update the literature findings, a series of interviews were conducted with the service providers.

**Step 2. Semi-structured interviews:** The goal of the interviews was to understand the broader context in which digital solution initiatives were being developed. What challenges do they face? what are the opportunities for future growth? We did not examine the technical details of individual innovations. The interviews were guided by a semi-structured framework based on key thematic areas outlined in the interview guideline. These themes included: (i) the organizational background, (ii) innovation characteristics and service Models, (iii) climate and agricultural advisory services, (iv) enabling environment (e.g., infrastructure, policies, financing); (v) data use and harmonization, (vi) social inclusion (for data access and use) and (vii) future outlook for digital advisory services.

**Step 3. Country-specific reports:** Based on the literature review and semi structured interviews, a report was produced for each of the six countries, addressing the current state of climate and agronomic digital advisory services in their respective agri-food systems (Table 2). Each report focused on the following four key topics:

**Table 2:** Description of digital agricultural solutions considered for review in WCA.

Digital AGricultural solutions (DAGs)	Service category	Service
Agronomic	Advisory	Farm operation, Storage, Input application
	Extension	Extension
	Identification of P&D	On-farm, Off-farm, Remote
Agricultural service providers	On-farm operation	Ploughing, Harvesting
	Off-farm operation	Transport, Storage
Climate	Climate information	Rainfall, Wind, Pests, Seasonal
	CSA	Seed, Ploughing system, Water management, Fertilizer, Cropping system, all
Crop insurance	Risks	Drought, Flood, Pest, Fire
Market information	Inputs	Seed, Fertilizer, Pesticide
	Outputs	Price, Quantity, Contacts off-takers
Finance	Input-credit	Seed, Fertilizer, Pesticide
	Investment	Investment
	On-farm operation	On-farm operation
	Output-credit	Output-credit
	Financial education	Financial education
Other DAGs	Others	Others

- *State of the art of existing services*: This section details the type of information provided by the digital services (e.g., weather forecasts, soil analysis, crop management advice), the frequency and format of the data (SMS, USSD, web-based platforms), and the analytical techniques used. Additionally, we examined the financial models sustaining these services identifying whether they were donor-funded, government-supported, or market-driven. The reports also analyzed the current and potential end-users of these services, including farmers, extension agents, service providers, and policymakers. The report also assessed the existing and future demand for such services.
- *Impact on Decision-Making*: The reports further explored how digital services contribute to better decision-making in terms of climate adaptation and resilience. The reports looked at the capacity of institutions to administer, analyze, and use the data from these services for decision-making in agri-food systems.
- *Challenges and Barriers*: A critical section of each country report was dedicated to identifying the main challenges associated with the implementation and scalability of climate and agronomic digital advisory services.
- *Potential of Digital Services and Value Chains*: The reports also highlighted the digital services and value chains with the greatest potential for growth and scalability. This analysis was based on factors such as market demand, institutional readiness, and the ability to integrate digital solutions into existing agricultural practices.

**Step 4. Report synthesis and recommendations:** Finally, the individual country reports were synthesized to provide a broader understanding of the regional landscape. The synthesis identified common trends, key barriers, and cross-country opportunities for collaboration and scaling digital services. Based on these findings, a set of recommendations was developed for stakeholders, including government institutions, the private sector, and development partners, to enhance the adoption and effectiveness of climate and agronomic digital advisory services.

## 3. Results

### 3.1. Projected climate change, agricultural impacts, and vulnerability

**Fig 3:**
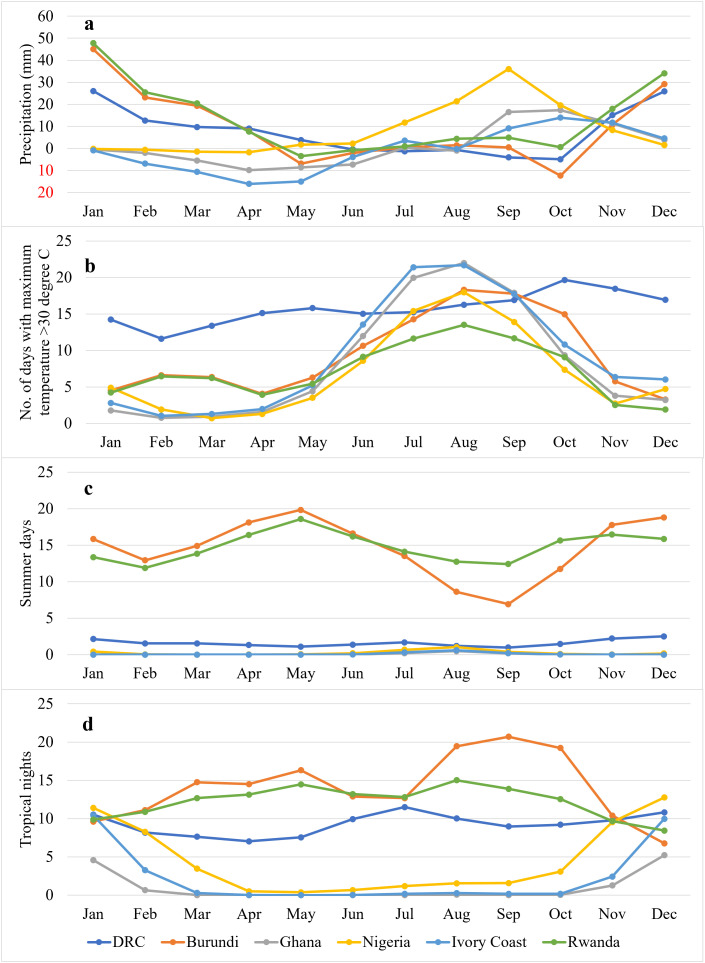
Trend of extreme indices by the end of the century in six study area countries: (a) precipitation; (b) number of days when maximum temperature is greater than 30° celsius; (c) summer days: No. of days when maximum temperature > 25° celsius; and (d) tropical nights: No. of days when minimum temperature > 20° celsius (Data source: World Bank).

Global warming is expected to increase climate variability, with more hot days, higher temperatures, and intense rainfall. Some areas may experience shifts in rainfall timing. Changes in extreme temperature and precipitation indices in the study area are depicted in Fig 3. Climate projections by the end of the century indicate higher flooding risks in Rwanda, Ghana, and Nigeria during the rainy season. Nigeria’s monthly rainfall will increase year-round, peaking in August to October (32 − 38% increase). Ghana will see more rainfall from September to January and less in other months. Ivory coast is expected to have a significant decrease in rainfall from February to May, possibly causing water shortages during these months. Rwanda will experience both an increase and a decrease, with a 20 − 32% rise between December to February. Overall, these changes will negatively impact the water balance across these countries.

Given these projected changes, the agricultural sector—dominated by smallholder, rainfed farming—will face significant challenges. Prolonged heatwaves and dry spells will likely lower water levels, reducing crop and livestock productivity and increasing mortality. Crop responses to changing climate vary. Maize yields are expected to decrease in Burundi, DRC, Ghana, and Ivory coast, but increase in Rwanda and Nigeria. Rice yields may increase in the DRC but decrease in Ivory coast, Rwanda, and Nigeria (IPCC, 2007). Areas with more rainfall could face pest and disease issues. Cassava, suited for hot and dry conditions, may suffer from waterlogging or reduced water availability. Livestock production is also at risk due to heatwaves and desertification.

**Fig 4:**
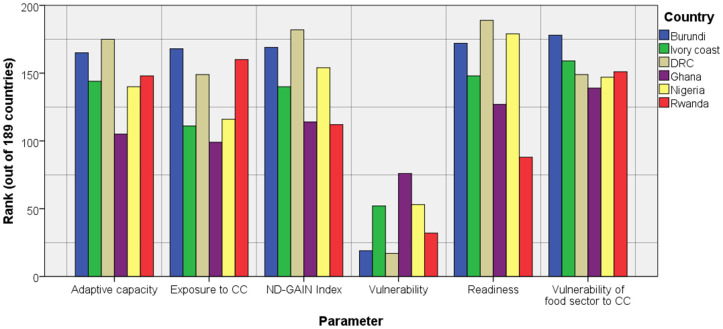
Climate vulnerability ranking of countries by notre dame-global adaptation index (nd-gain 2023) [14].

Due to the ongoing and expected climate change, most of these countries were identified as highly vulnerable to climate change and least ready to combat its ill-effects ( [14], Fig 4). In the ND-GAIN ranking, the DRC followed by Burundi ranked higher in terms of vulnerability (where a higher rank means higher vulnerability), whereas the two countries along with Nigeria ranked lower in terms of readiness as well as adaptive capacity (a lower rank indicates poor adaptive capacity). Among the six African countries examined, Ghana exhibits relatively higher exposure to climate change; however, its relatively strong adaptive capacity as well as readiness makes it the least vulnerable.

### 3.2. Existing climate and agronomic digital solutions

#### 3.2.1. Overall status of digital agricultural solutions (DAGs).

**Fig 5:**
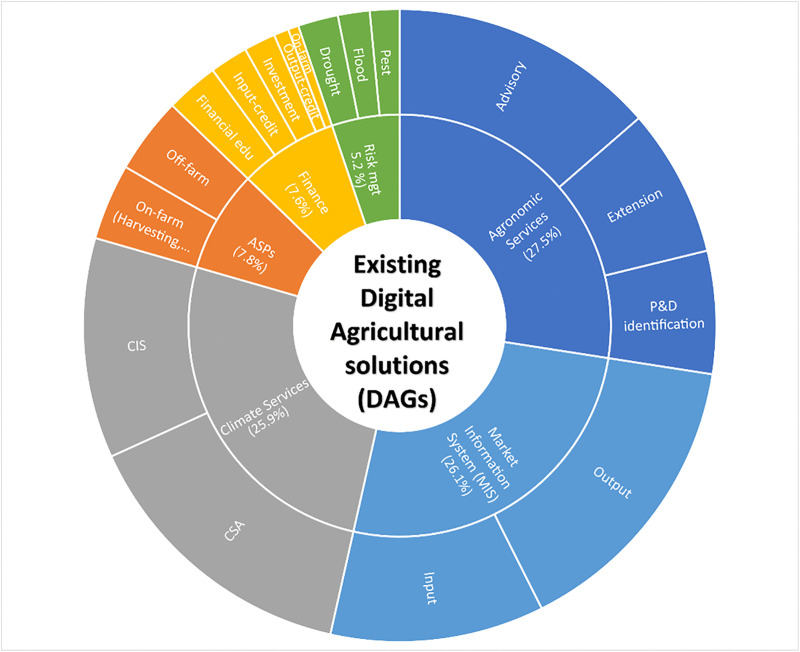
Existing digital agricultural solutions (DAGs) in the study area. ASPS: Agricultural Service Provider; CIS: Climate Information Services; CSA: Climate Smart Agriculture; P&D: Pest and Disease.

Each of the six study countries has developed diverse solutions to provide climate and agronomic advice to farmers. Many of these services rely on mobile-based platforms, such as SMS and USSD, to disseminate weather forecasts, agronomic advice, and information about the market. Fig 5 shows major DAGs available in the study area’s six countries. DAGs focused on agronomic practices, market information and climate services together hold a major share (~80%) of the initiatives in the study area. In agronomic services, advisory and extension services seem to be a priority (Fig 5). Pest and Disease (P&D) identification services were reported in greater numbers from Ghana, Nigeria, and Rwanda, and relatively fewer in other countries.

In terms of market information, a greater emphasis was placed on output-related information (58%), particularly off-taker contacts and output prices, with relatively less focus on inputs such as seeds. Digital solutions addressing climate change were mainly offered as Climate Information Services (CIS) and Climate Smart Agriculture (CSA) practices. CIS typically provide information on rainfall, wind, pests, and seasonal forecasts, while CSA solutions covered cropping systems, fertilizers, seeds, ploughing, water management, and other adaptation measures combined. Overall, most climate-related innovations (57%) focus on CSA, with 43% targeting CIS (Fig 5). Relatively fewer DAGs were reported with respect to Finance, Agriculture Service Providers (ASPs), and risk management, indicating notable service gaps in the study area.

The country-wise distribution of the types of DAGs, however, shows that these figures varied significantly between the countries (S1 Fig). Ghana leads in agronomic services across advisory, extension, and P&D identification, followed by Rwanda and Nigeria, while Burundi and the DRC report the fewest (S2 Fig). Climate services also reflect contrasting priorities: Nigeria, Ghana, and Rwanda place greater emphasis on CSA, whereas Burundi, Ivory coast, and the DRC focus more on CIS (S3 Fig). Innovations in Ghana, Nigeria, and Rwanda often provide comprehensive CSA packages, while those in the other three countries typically address individual CSA components. For example, Burundi devotes 25% of its activity to water management, Ivory coast emphasizes cropping systems and seeds, and the DRC focuses more on seed and fertilizer solutions (Table 3). CIS innovations are widespread, with Ghana, Nigeria, and Rwanda prioritizing seasonal forecasts, while the DRC leans more on rainfall and wind information. Pest forecasts, however, are available across all six countries. About 93% of services were available throughout the crop growing period, 5% seasonally, and the rest annually.

**Table 3:** Proportion of innovations (%) providing different digital climate services in WCA.

Country	CSA						CIS			
	All CSA	Cropping system	Fertilizer	Ploughing system	Seed	Water mgt	Rainfall	Seasonal	Wind	Pest
Burundi	0.0	18.8	18.8	18.8	18.8	25.0	38.1	4.8	33.3	23.8
Ivory coast	0.0	30.8	15.4	15.4	23.1	15.4	42.9	0.0	28.6	28.6
DRC	0.0	16.7	33.3	8.3	25.0	16.7	36.4	0.0	40.9	22.7
Ghana	42.9	16.7	14.3	7.1	7.1	11.9	20.0	46.7	0.0	33.3
Nigeria	47.2	19.4	8.3	2.8	11.1	11.1	0.0	87.5	0.0	12.5
Rwanda	58.8	0.0	23.5	0.0	0.0	17.7	20.0	60.0	0.0	20.0
**% of grand total**	**33.1**	**16.9**	**16.2**	**7.4**	**11.8**	**14.7**	**31.7**	**20.2**	**23.1**	**25.0**

Market information services also showed uneven distribution. Nigeria, Ivory coast, and Ghana together accounted for around 60% of such innovations (S4 Fig), while innovations related to credit, ASPs, and risk management were much fewer across all countries (S5–S7 Figs). This gap is particularly concerning for Ghana, the DRC, and Rwanda, given their strong agricultural growth potential. Overall, Ivory coast, Ghana, and Nigeria contributed about 20% each of total innovations, whereas Burundi, Rwanda, and especially the DRC had fewer. In Rwanda and Burundi, this is partly due to their small size and challenging topography, while in the DRC, the limited number highlights a need for greater investment and development. The review also found out that most innovations were launched only in the past decade (result not presented). The private sector has played a dominant role, accounting for more than 82% of services, while the public sector contributed only about 4%, underscoring both the rapid expansion of market-driven solutions and the limited public sector engagement in scaling DAGs.

#### 3.2.2. Users of digital agricultural solutions (DAGs).

This study identified three different user groups of digital agro-climatic technologies and advisory services: farmers, service providers, and policy makers. Each user group differs in terms of their needs, the type of services they use, their motivation for accessing their services, and the main barriers they face in accessing these services. In this paper, we only discuss farmers as users of these services.

**Fig 6:**
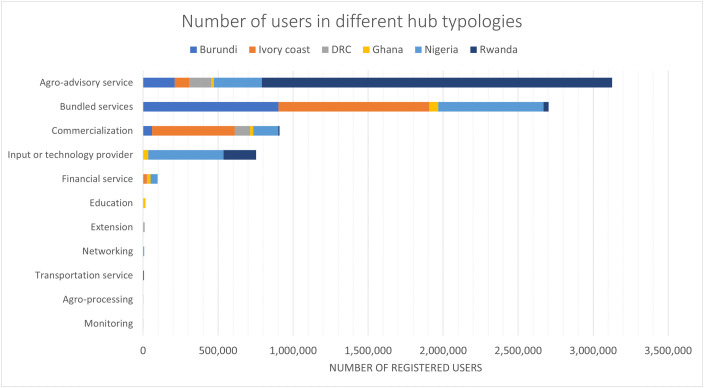
Number of users registered in different hub typologies.

Farmers were the primary beneficiaries of agricultural digital technologies, with diverse needs such as tailored agro-advisory services, real-time climate information, better access to insurance, financial services, market opportunities, inputs, mechanization, and post-harvest storage. Fig 6 presents the diverse range of hub types and their varying prominence across different countries. The top four hub typologies in terms of registered users—agro-advisory services, bundled services, input and technology providers, and commercialization—highlight a focus on agricultural development. These services provide integrated solutions, technological advancement, and improved market accessibility across countries, driving economic growth and sustainability. While Rwanda stands out as having more users for agro-advisory services, Ivory coast followed by Burundi and Nigeria excels in bundled services. Although input and technology provider have a relatively smaller user base, these services were prominent in Nigeria followed by Rwanda. Financial services were provided mainly in Ivory coast, Ghana and Nigeria.

The review showed a high level of awareness and adoption of digital technologies among farmers, except in Ghana and DRC. In total, Rwanda had the highest number of registered users benefiting from these services, followed by Nigeria and Ivory coast (S8 Fig). However, farmers faced challenges in using the tools effectively due to limited capacity. They encounter barriers such as limited technology ownership, low payment ability, low literacy, and lack of support services.

#### 3.2.3. Existing tools and challenges in complementing dags.

**Extension and Research Services:** Across the six study countries, several tools and initiatives have been developed to strengthen agricultural value chains and improve access to DAGs. Public extension and research systems remain central, though they vary in structure. Nigeria has the T*raining and Visiting scheme* [15], while Rwanda operates *Twigire Muhinzi* [16], a decentralized model combining Farmer Field Schools and Farmer Field Promoters. Nigeria also hosts the IITA and the largest agricultural research system in Sub-Saharan Africa. These systems have expanded farmer outreach but face severe capacity constraints: the extension worker-to-farmer ratio is 1:7,500 in Nigeria and 1:1,500 in Ghana, far above FAO’s 1:500 recommendation [17]. Women remain underrepresented among extension agents (5–28% of total extension personnel), limiting technology dissemination in settings where sociocultural norms constrain interactions between men and women [18,19]. Declining public funding has shifted reliance toward NGOs and the private sector [4,20–22], though public officers still form the majority of extension staff [23]. Persistent challenges include inadequate ICT tools, limited training, weak infrastructure, and gender disparities, though recent reforms aim to modernize systems by increasing private sector participation and prioritizing cash crops.

**Policy Landscape:** The policy environment has also advanced digital agriculture. Ghana, after Kenya, has taken the lead in ICT regulation, with economic liberalization attracting foreign investment in digital infrastructure, particularly in telecommunications [24]. Over the past decade, governments have launched programs to promote ICT in agriculture, including creating digital identities of farmers, implementing e-payments, transferring technology, delivering inputs, sharing weather and market data, diversifying the economy, and supporting import substitution strategies in selected value chains. Yet challenges remain in selecting beneficiaries, ensuring timely input supply, and generating data for monitoring and evaluation [25]. Public investment in agriculture is generally low (0–2.5% of budgets), except in Rwanda [4], and has even declined in Nigeria. No reports were available for Burundi, Ivory coast, or the DRC. Staffing shortages further limit policy implementation capacity; for example, Ghana’s agricultural agencies operate with less than half of required staff [3].

**Business Environment:** On the business side, liberal policies and foreign investment have stimulated the creation of tech hubs, innovation centres, incubators, and accelerators, many supported by CGIAR and NGOs [26]. These have fostered entrepreneurship in agriculture and climate-related solutions. However, access to finance remains a major constraint, particularly for smallholders. Limited agricultural investment, weak credit access, and repayment challenges undermine farmers’ capacity to adopt DAGs. Crowdsourcing has emerged as an alternative financing model, though still at a small scale.

**Data Harmonization and Data Management**: Unlike many similar exercises, this mapping study includes a focus on data harmonization and data management, highlighting their importance for effective digital advisory services. The findings indicate that in DRC, data is stored independently by various ASPs, and there is little to no coordination between them in terms of data harmonization. The absence of a common platform for data storage and integration complicates efforts to create a standardized system for managing agricultural data. Metadata is often missing, further impeding data sharing and interpretation. Burundi faces similar challenges, lacking a centralized repository for data. Ivory coast struggles with the absence of a unified data harmonization framework, hindering data sharing and consolidation. Ghana has made progress with the National Data Sharing Policy and efforts to integrate agricultural data, though harmonization is still in early stages. In Nigeria, gaps in the regulatory framework regarding data privacy and harmonization limit the potential of digital agricultural data sharing. Rwanda has made progress with the Rwanda Open Data Portal, but data integration remains fragmented and requires further development for seamless sharing. In summary, the lack of data harmonization makes it difficult to optimize the use of digital services for decision-making in agriculture.

**Challenges:** Despite these enabling tools, significant challenges continue to constrain digital agriculture.

- Infrastructure deficits—especially electricity, roads, and network connectivity—remain the greatest bottleneck, with sharp rural-urban divides [27]. High internet costs compound the problem: in Rwanda, fixed broadband subscription costs about 45.7% of household expenditure, while mobile data accounts for 7.8–11.1% [28]. Although mobile coverage is extensive, only 69% of the population uses the internet, with men’s usage far higher than women’s.
- Low literacy and digital skills further restrict adoption of DAGs. Although adult literacy rates was more than 75% in most countries (except Nigeria), digital skills were ranked as the top barrier to mobile internet adoption [29]. Computers remain unaffordable for most rural households, and basic reading and writing challenges hinder access to advisory content [30]. Disseminating information in local languages – either written or as voice or video records – were therefore suggested as a more inclusive approach.
- Gender and social inclusion gaps also remain critical [31]. Women constitute over one-third of the agricultural workforce and more than half in rural areas [32], yet they face unequal access to land, finance, and technology. Women represent only about a quarter of digital technology users in Sub-Saharan Africa [33], limiting their access to advisory services [19,34]. Lack of communication assets and social group participation also reduces their access to climate information [35,36].
- Data harmonization and management remain major challenges across the study countries, with fragmented systems, missing metadata, and weak regulatory frameworks limiting interoperability (Table 4). In most countries, significant gaps persist: the DRC and Burundi lack centralized repositories, Ivory coast has no harmonization framework, and Nigeria faces regulatory shortcomings in data privacy and integration. Although Ghana’s National Data Sharing Policy and Rwanda’s Open Data Portal show initial progress, both remain at early or fragmented stage. These gaps constrain effective data sharing and reduce the potential of digital services for decision-making in agriculture.
- Finally, lack of trust across value chains—including contract breaches, unfair pricing, and corruption—discourages adoption of digital solutions. To address this, some tools have introduced certification and traceability systems, blockchain for land and financial transactions, and platforms for improved information sharing.

**Table 4:** Challenges identified in complementing digital agriculture solutions.

Country*	Costs of services	Data harmonization	Data privacy	Digital literacy	Languages	Local capacity
Ghana	35	24	27	28	36	35
Nigeria	31	26	32	36	39	29
Rwanda	23	15	19	18	19	25

*Information not available for Burundi, Ivory coast, and the DRC

In summary, while extension systems, supportive policies, and business innovations provide a growing toolkit for digital agriculture, their effectiveness is undermined by systemic barriers. These include weak infrastructure, high connectivity costs, limited digital literacy, gender and social inequalities, financing gaps, and low trust within value chains. Overcoming these challenges will be crucial for scaling climate and agronomic digital advisory services equitably and sustainably across the study countries (Table 4).

#### 3.2.4. Existing dissemination tools.

The spread of digital agricultural services has been made largely through bundled services. Review suggests that these services were made available by combining CIS-CSA based agro-advisory with other services, such as (1) telephony services (such as ESOKO, VIAMO and FarmerLine); (2) logistics, value chain traceability and payment services (Caargil and FarmerLine); (3) financial and insurance services (AgUnity and AgroCenta); (4) electronic services for financial transactions; and (5) the logistics solutions for access to post-harvest infrastructure. Bundled services have been a model that has been able to meet various needs of a target user by complementing their offer with complementary services.

## 4. Discussion

This study presents the evolving landscape of digital agricultural solutions (DAGs) across six countries in WCA. The findings underscore both the urgency of adapting to intensifying climate risks and the opportunities that digital innovations offer with respect to enhancing agricultural productivity, climate resilience and decision-making for smallholder farmers in the study area. The integration of near-real time, accurate and personalized agricultural information—especially weather forecasts, pest alerts, market information, and agronomic advice—enables farmers to plan farm operations more effectively and reduce risks associated with extreme weather events, pests, and diseases [37–39]. Crop insurance services help farmers manage financial risks by providing protection against losses from extreme weather, pests, and diseases. A recent study investigated the ways in which public climate adaption planning in sub-Saharan Africa can be aided by climate risk information generated in the context of insurance-related operations [40]. The authors found that although there is risk awareness and a general intent to manage climate risks, the main obstacles were unclear risk ownership, limited trust, and/or a lack of incentives.

### 4.1. Digital agricultural solutions as an adaptation pathway

Digital innovations in agriculture sector offers a promising pathway to enhance climate resilience [41]. Agricultural extension and advisory services (EAS) have undergone rapid digitalization over the past decades [42]. The rapid growth of DAGs across the study countries, and the dominance of mobile-based platforms for disseminating climate and agronomic information illustrates the transformative potential of digital tools in bridging information gaps for farmers. Digital innovations bring new opportunities for EAS delivery, including improving accessibility to services in geographically remote areas, bridging the information gaps among different value chain actors, and contributing to fair trade and inclusive markets, social and financial inclusion and others [42]. The high registration rates for agro-advisory and bundled services in the study area reflect their perceived value and growing user demand.

The predominance of innovations in agronomic, market information and climate services reflects the agriculture sector’s vulnerability to climate risk [13,43] and increasing awareness about climate adaptation. However, the uneven distribution of services across the countries indicates significant service disparities, i.e., farmers in Burundi, Ivory Coast, and the DRC may remain underserved relative to those in Ghana, Nigeria, and Rwanda. The predominance of private-sector-led initiatives (82%) further suggests that while market-driven approaches are driving innovation, public sector involvement remains limited. This raises concerns about inclusivity, as resource-poor farmers and marginalized groups may be bypassed without stronger public sector engagement. This imbalance may constrain the long-term scalability and inclusivity of digital agriculture, especially for marginalized groups who may be less attractive to private investors.

Ofosu-Ampong et al. 2025 reviewed a range of Digital agro-advisory tools in the global south and their adoption rate [44]. The authors found CSA, farmer empowerment as the top priority, followed by financial inclusion and EWS as having a medium adoption rate. Tools providing services related to information dissemination and post-harvest loss reduction were classified as low-adoption tools. Similar to our study, digital literacy, inadequate infrastructure, along with insufficient human capital development and policy environments were identified as the key constraints for smallholder farmers to access and benefit from digital EAS [44,45].

### 4.2. User adoption and barriers

Although awareness and adoption of digital tools are increasing, several barriers continue to limit effective uptake. Limited digital literacy, high service costs, poor connectivity, low trust in service providers, and socio-economic exclusion constrain the reach and impact of DAGs [44,45]. Gender disparities are particularly critical, as women—though integral to agricultural production—have disproportionately lower access to digital tools, which reflects broader structural inequalities in land access, finance, and technology ownership. Weak extension services further restrict farmers’ ability to integrate digital information into practice, with farmer-to-extension worker ratios far exceeding FAO recommendations. The lack of integration between public and private initiatives exacerbates inefficiencies, as does the absence of harmonized agricultural data systems. In countries like the DRC and Burundi, fragmented data collection reduces opportunities for coordinated planning, while regulatory gaps in Nigeria hinder data privacy and interoperability. Without strengthening these enabling systems, the transformative potential of DAGs will remain underutilized.

The exclusion of end-users—particularly farmers—from the design and development process further compounds the issue. The use of Human-Centered Design (HCD) approaches, which involve co-creation with farmers, service providers, and policymakers, offers a promising pathway to enhance usability, accessibility, and relevance. HCD also enables better integration of climate data into business models and policymaking platforms, while ensuring services are tailored to local language and context. Similar to our study, inclusive design and requirement elicitation were found essential to ensure that these tools are accessible and relevant to the needs of smallholder farmers [44]. This review emphasizes that for DAGs to be effective, inclusive design must be prioritized. Gender, age, education, and regional disparities should be central considerations in platform development and outreach strategies. Failure to do so risks exacerbating existing inequalities in access to agricultural services.

### 4.3. Opportunities and policy implications

Despite several challenges, the results point to several promising opportunities and investment priorities. Our findings suggest that many services remain narrowly focused—targeting specific crops, inputs, or farmer groups. This fragmentation often forces farmers to engage with multiple platforms, increasing costs and complicating access. Bundled services, which combine agro-advisories with essential services like credit, insurance, and access to inputs and market, appear to be especially effective in addressing multiple farmer needs simultaneously [46]. This approach minimizes transaction costs for farmers and increases efficiency for providers [19,46,47]. Present review revealed that bundled services came next to the agro-advisory services in terms of the number of registered users in the study area, suggesting high awareness as well as their demand.

However, operationalizing such models requires strengthening rural infrastructure and connectivity, promoting digital literacy through inclusive training, and ensuring gender-responsive approaches. Public-private partnerships can help scale successful innovations while addressing gaps in access and affordability. In rural areas, where these prerequisites are often lacking, the scalability and sustainability of digital advisory platforms remain constrained. Addressing these foundational issues is essential for service equity and long-term adoption. Finally, improved data governance, including harmonization and privacy safeguards, are critical for maximizing the value of digital platforms. Addressing these systemic constraints will determine whether digital agriculture evolves into a tool for inclusive climate adaptation or exacerbates existing inequalities.

Furthermore, national policy frameworks play a vital role in shaping the digital agriculture landscape. Countries such as Rwanda and Nigeria have implemented progressive policies, including the National Agricultural Technology and Innovation Policy (2022) and Digital Agriculture Strategies, which aim to promote youth engagement, simplify enterprise operations, and improve access to land, credit, and training [48]. These measures have facilitated greater participation among younger, technologically up-to-date farmers—an important demographic for the uptake of digital tools [49,50]. Nonetheless, significant gaps remain in data governance. While open data (e.g., crop, weather, and market data) is key to innovation, limited cross-organizational collaboration and inconsistent regulatory frameworks restrict access—particularly for private sector actors and emerging agri-tech start-ups. Issues of data privacy, ownership, and cybersecurity also persist. Although countries like Ghana, Nigeria, and Rwanda have initiated legislative responses, others—such as Burundi and Ivory coast—lack adequate policies for agricultural data governance. The absence of harmonized and interoperable systems further constrains the scalability and integration of digital services.

### 4.4. Limitations and future research

While this review mapped a diverse range of existing digital solutions, the analysis is limited by data availability, particularly for Burundi, Ivory Coast, and the DRC. Furthermore, the study primarily examined farmer adoption, leaving gaps in understanding how service providers and policymakers utilize these tools. Future research should focus on assessing the effectiveness of DAGs in improving agricultural productivity under climate stress, with attention to gender and equity outcomes, considering the size of country. Longitudinal studies would also help to evaluate whether current innovations deliver sustained resilience over time.

In summary, these findings indicate that while digital agriculture is rapidly expanding, its potential to build climate resilience is constrained by deep structural and institutional barriers. Closing these gaps will require coordinated investments in infrastructure, digital literacy, and inclusive policy frameworks, alongside efforts to harmonize data and strengthen governance. Moreover, ensuring that digital solutions are accessible to marginalized groups, particularly women and rural smallholders, will be critical for achieving equitable adaptation outcomes. Without such measures, DAGs risk reinforcing existing inequalities rather than serving as tools for resilience and transformation.

## 5. Conclusion

This study demonstrates that digital agricultural solutions (DAGs) hold significant potential to strengthen climate resilience and agricultural productivity in West and Central Africa, particularly through mobile-based advisories, climate-smart agriculture practices, and bundled service models that integrate market, financial, and climate services. These tools can reduce smallholder exposure to climate risks by improving access to timely information, resources, and decision support. However, major barriers—including poor infrastructure, high connectivity costs, low digital literacy, and gender disparities—continue to constrain equitable adoption. The predominance of private-sector initiatives has fueled rapid innovation but raises concerns about inclusivity and long-term sustainability without stronger public sector involvement and supportive policy frameworks. Unlocking the full potential of digital agriculture will require targeted investment in rural infrastructure, inclusive training, and gender-responsive approaches, alongside improved public–private collaboration and harmonized data systems. Future research should move beyond mapping existing tools to rigorously assess their long-term impacts on productivity, equity, and resilience, with particular attention to gender and youth inclusion. Integrating DAGs into broader adaptation strategies that connect technology, institutional capacity, and inclusive policies will be essential to ensuring they contribute to sustainable and equitable food systems under a changing climate. This synthesis paper also highlights the diverse needs and challenges faced by the key user groups of DAGs, and proposes a Human-Centered Design (HCD) approach as a potential solution to improve adoption and impact of these services.

## Supporting information

S1 Fig Country-wise distribution of existing digital agricultural services in six countries of WCA. (PNG)

S2 Fig Country-wise distribution of innovations providing agronomic services in six countries of WCA. (TIF)

S3 Fig Country-wise distribution of innovations providing digital climate solutions in six countries of WCA. (TIF)

S4 Fig Country-wise distribution of innovations providing market information in the six countries of WCA. (TIF)

S5 Fig Country-wise distribution of innovations providing finance in the six countries of WCA. (TIF)

S6 Fig Country-wise distribution of innovations identified as agricultural service provider in the six countries of WCA. (TIF)

S7 Fig Country-wise distribution of innovations providing crop insurance to different risks in the six countries of WCA. (TIF)

S8 Fig Existing users of digital agricultural solutions in various value chains. (TIF)
