# Supplementary figures and images for: Mapping the climate and agronomic digital advisory services landscape in West and Central Africa: A step towards making food systems productive and climate resilient
Source: PLoS One. 2025 Dec 3;20(12):e0338010. doi: 10.1371/journal.pone.0338010 (PMC12674564; doi:10.1371/journal.pone.0338010)

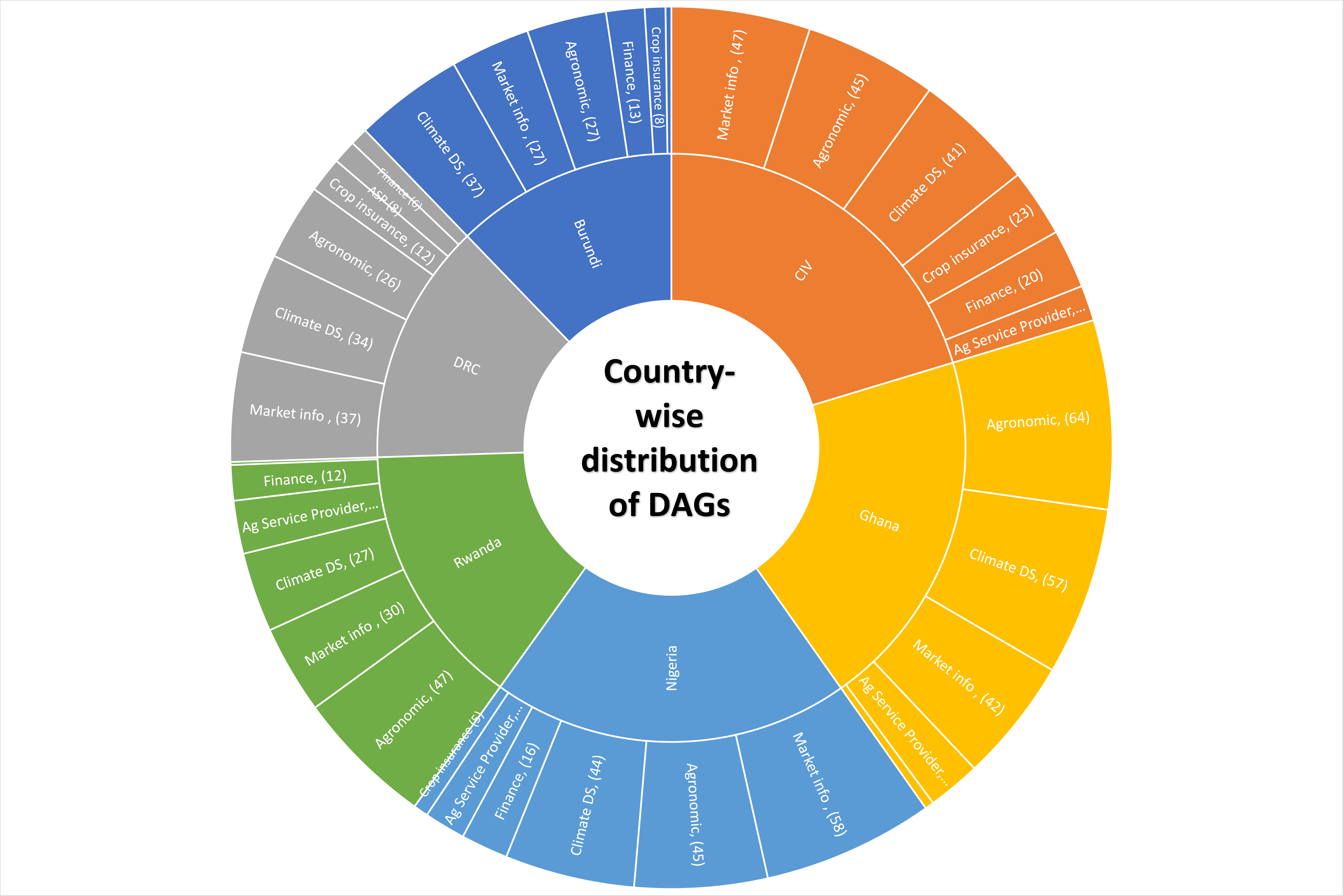

Supplement: S1 Fig — (PNG) [file pone.0338010.s001.png]

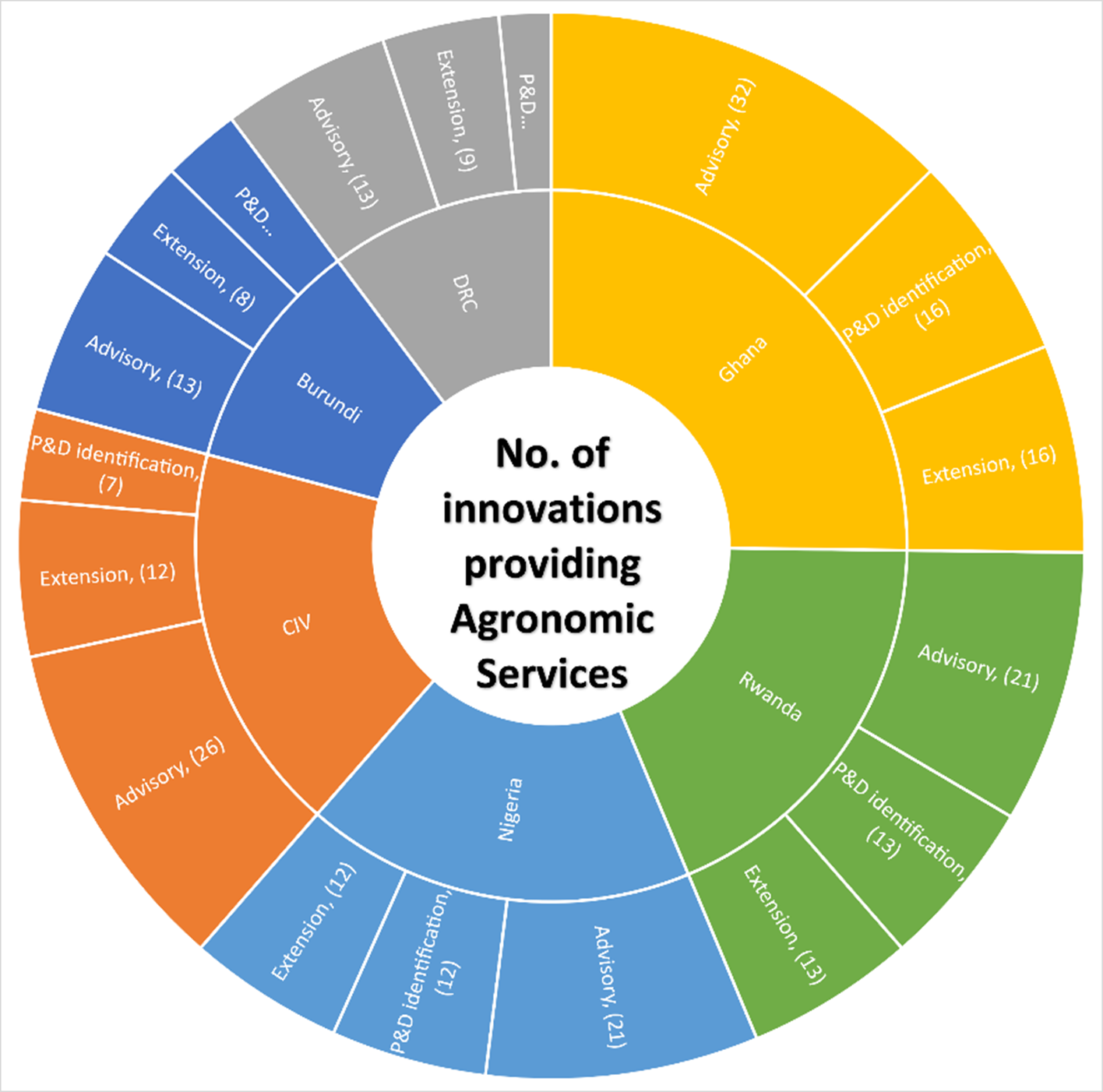

Supplement: S2 Fig — (TIF) [file pone.0338010.s002.tif]

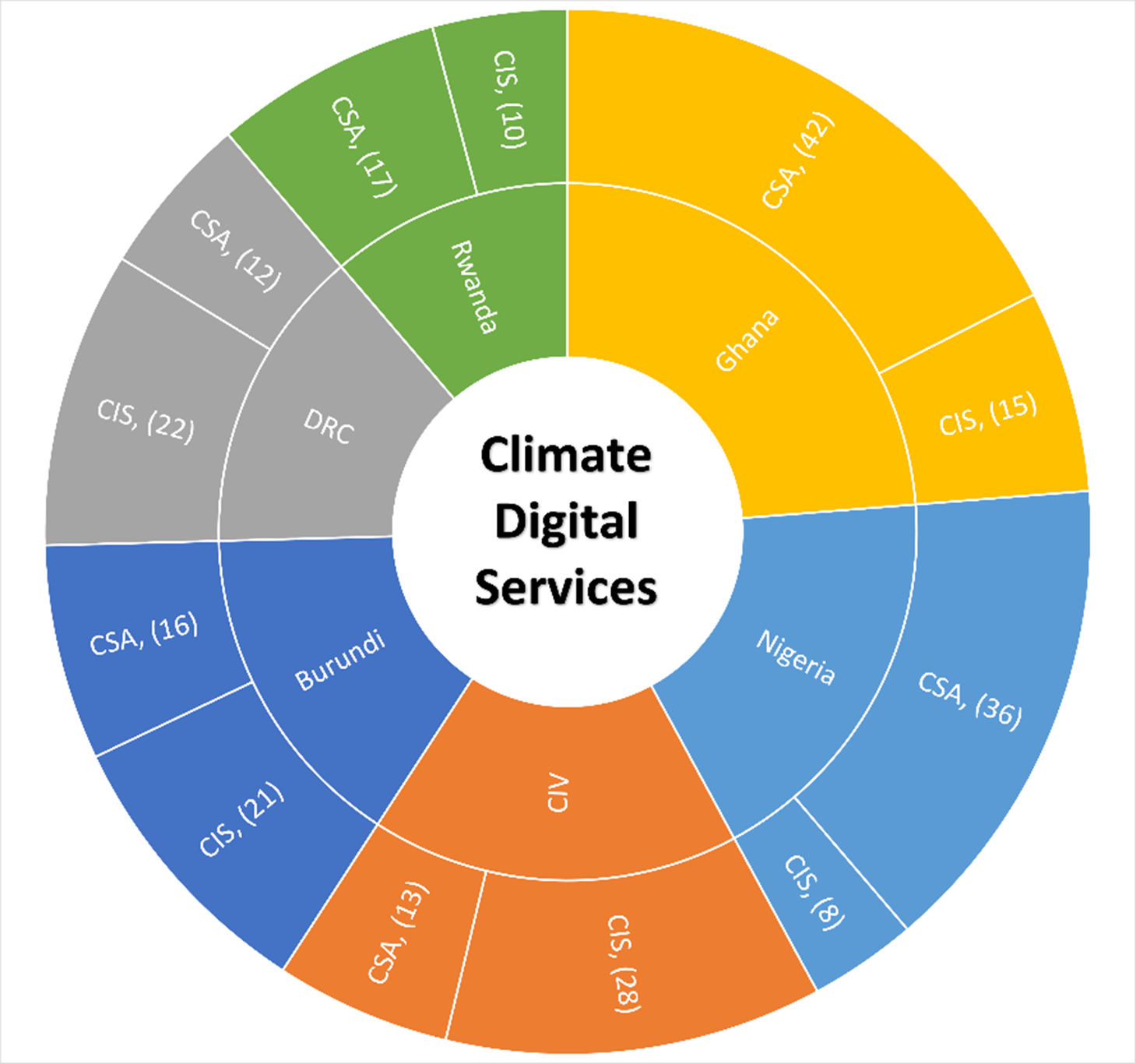

Supplement: S3 Fig — (TIF) [file pone.0338010.s003.tif]

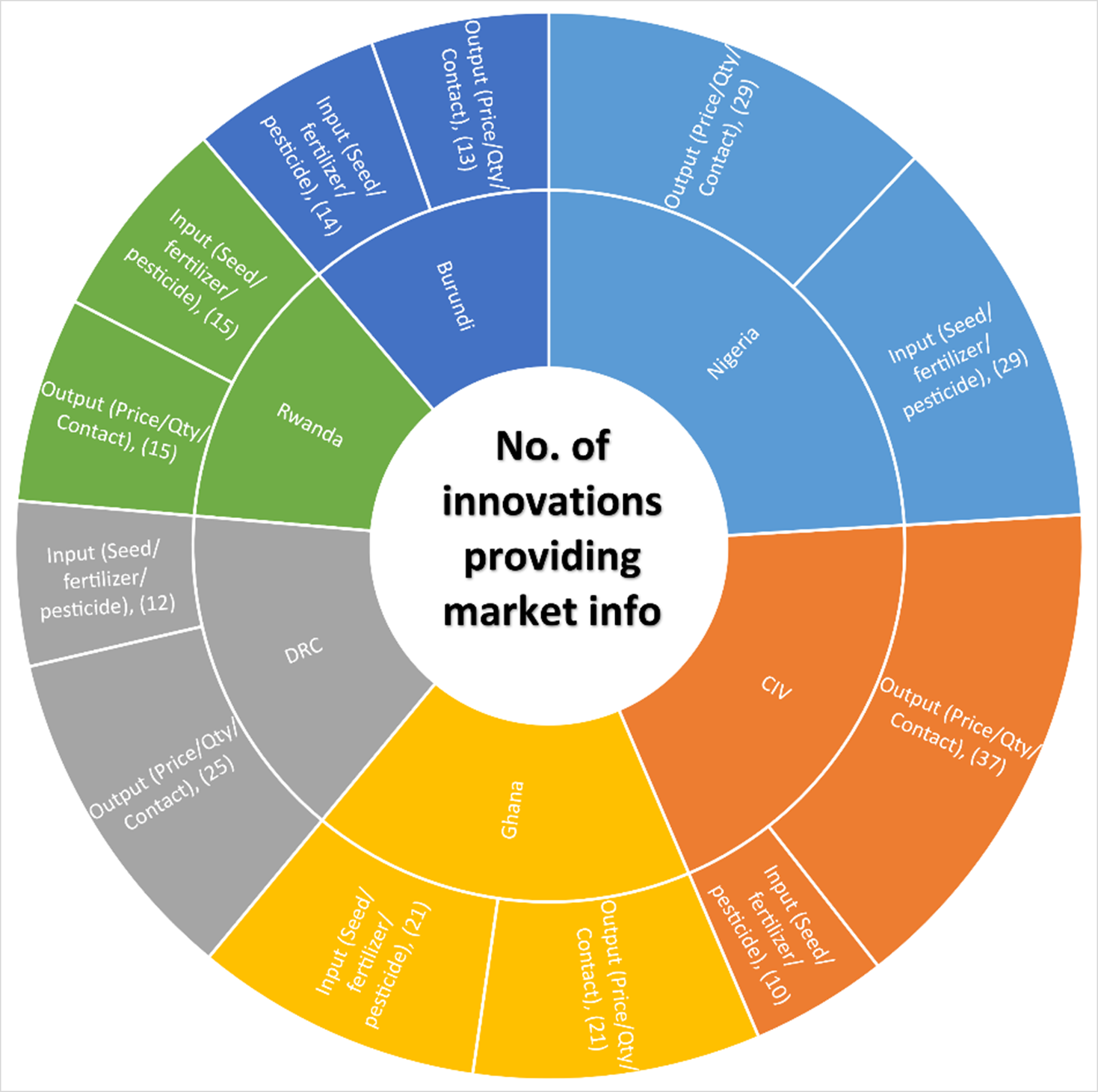

Supplement: S4 Fig — (TIF) [file pone.0338010.s004.tif]

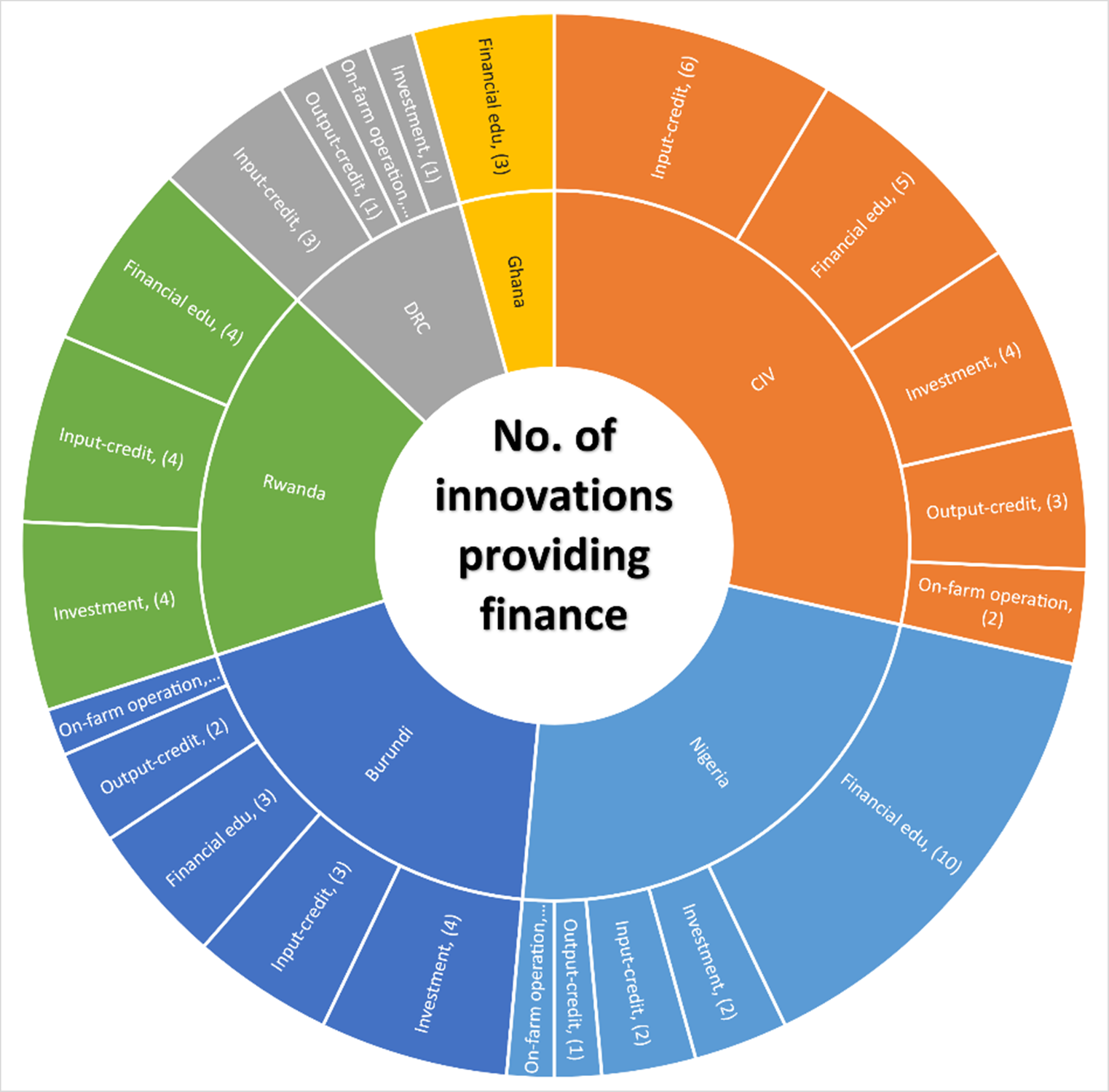

Supplement: S5 Fig — (TIF) [file pone.0338010.s005.tif]

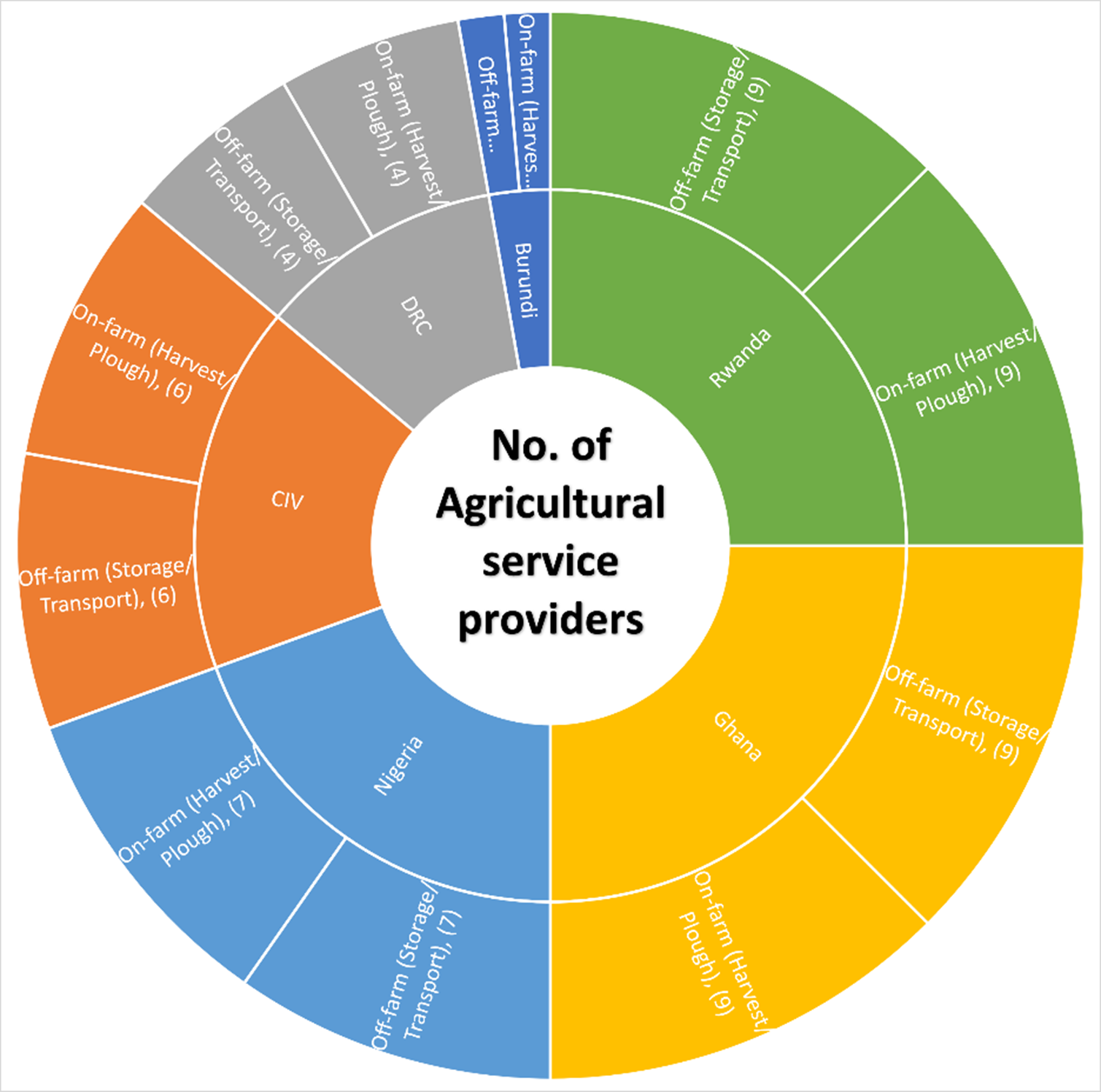

Supplement: S6 Fig — (TIF) [file pone.0338010.s006.tif]

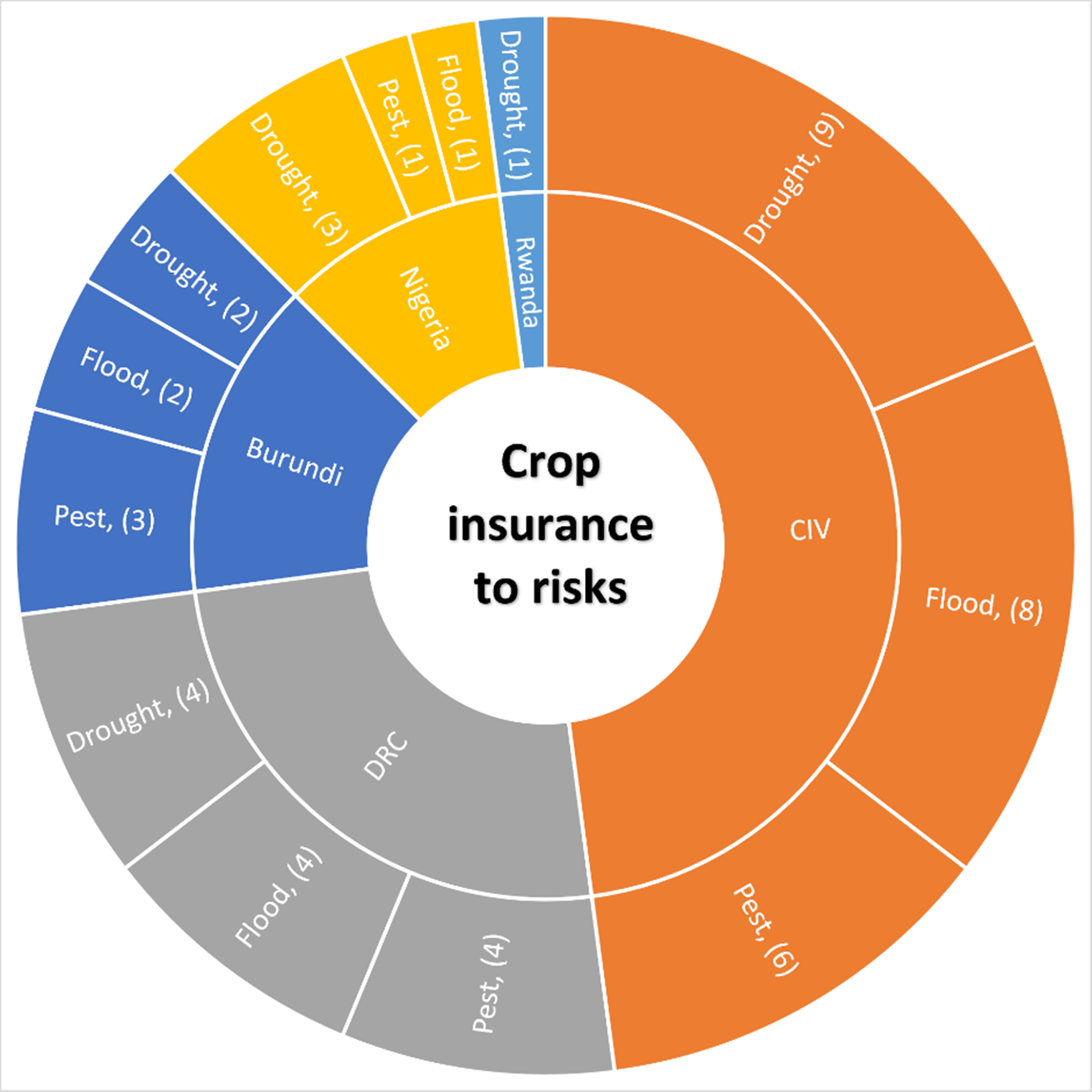

Supplement: S7 Fig — (TIF) [file pone.0338010.s007.tif]

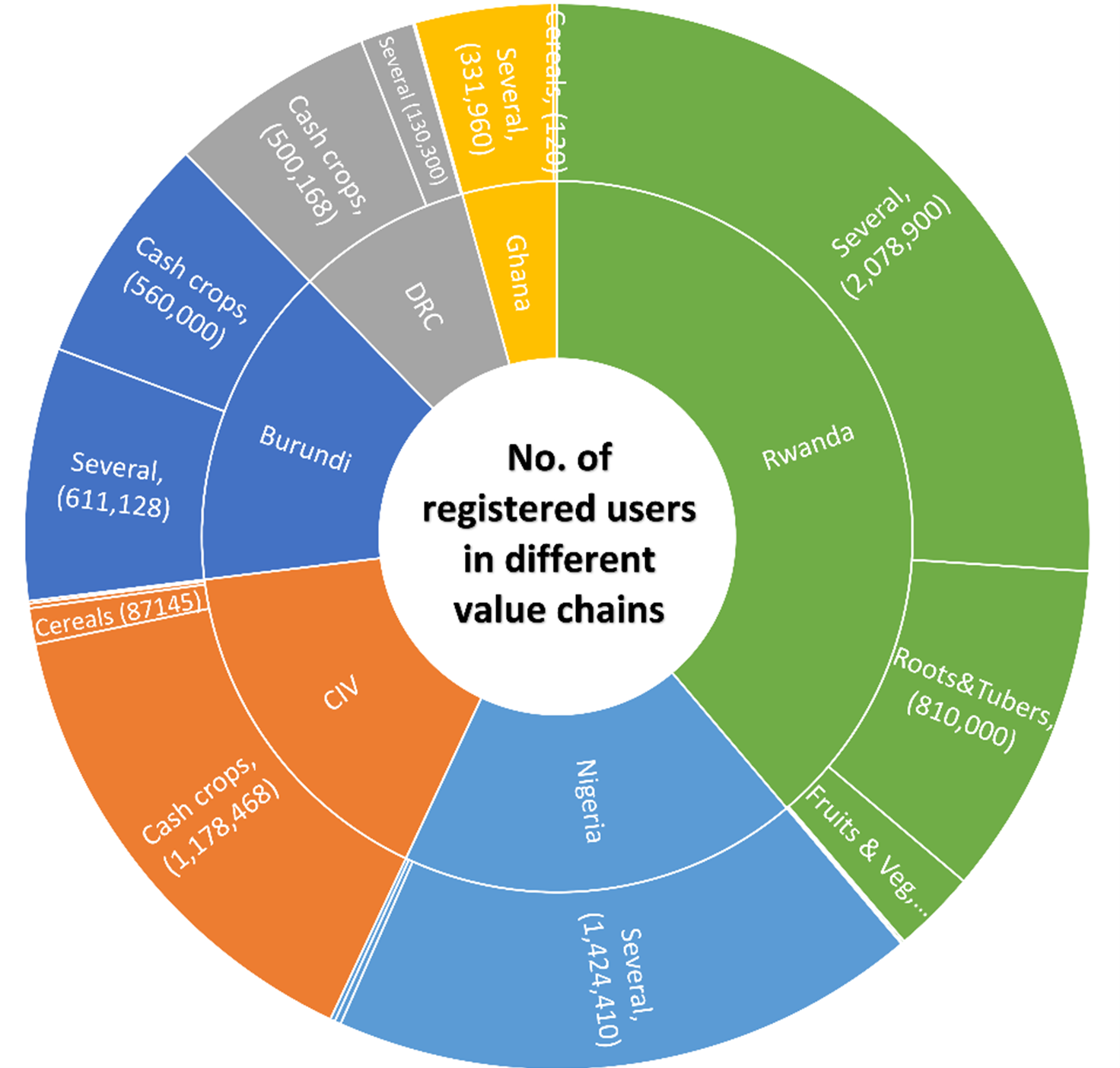

Supplement: S8 Fig — (TIF) [file pone.0338010.s008.tif]
